# Pelvic floor muscle strength of women consulting at the gynecology outpatient clinics and its correlation with sexual dysfunction: A cross-sectional study

**DOI:** 10.12669/pjms.334.12250

**Published:** 2017

**Authors:** Filiz Ciledag Ozdemir, Erkan Pehlivan, Rauf Melekoglu

**Affiliations:** 1 Filiz Ciledag Ozdemir, Department of Physical Therapy and Rehabilitation, Inonu University School of Medicine, Malatya, Turkey; 2 Erkan Pehlivan, Department of Public Health, Inonu University School of Medicine, Malatya, Turkey; 3 Rauf Melekoglu, Department of Gynecology and Obstetrics, Inonu University School of Medicine, Malatya, Turkey

**Keywords:** Pelvic floor muscles, Risk factors, Sexual dysfunction

## Abstract

**Objective:**

To investigate the pelvic floor muscle strength of the women andevaluateits possible correlation with sexual dysfunction.

**Methods:**

In this cross-sectional type study, stratified clusters were used for the sampling method. Index of Female Sexual Function (IFSF) worksheetwere used for questions on sexual function. The pelvic floor muscle strength of subjects was assessed byperineometer. The chi-squared test, logistic regression and Pearson’s correlation analysis were used for the statistical analysis.

**Results:**

Four hundred thirty primiparous women, mean age 38.5 participated in this study. The average pelvic floor muscle strength value was found 31.4±9.6 cm H_2_O and the average Index of Female Sexual Function (IFSF) score was found 26.5±6.9. Parity (odds ratio OR=5.546) and age 40 or higher (OR=3.484) were found correlated with pelvic floor muscle weakness (p<0.05). The factors directly correlated with sexual dysfunction were found being overweight (OR=2.105) and age 40 or higher (OR=2.451) (p<0.05). Pearson’s correlation analysis showed that there was a statistically significantlinear correlation between the muscular strength of the pelvic floor and sexual function (p=0.001).

**Conclusion:**

The results suggested subjects with decreased pelvic floor muscle strength value had higher frequency of sexual dysfunction.

## INTRODUCTION

The pelvic organs are supported mainly by the muscular activity of the pelvic floor and its ligaments. The striated muscles of the pelvic floor play an important role in miction, defecation and sexual function. They provide dynamical support to the pelvic organs by adapting the muscular tension to changing conditions. Different mechanical, neural and hormonal factors cause a reduction of muscle strength in the pelvic floor, thus interfering with the supportive function of the musculature.^1^

Pelvic floor dysfunction is a term generally used for malfunctions of the continence mechanism and for pelvic organ prolapse. It causes physical, social and sexual problems that negatively impact quality of life.^2^

Sexual dysfunction is defined as the disturbance in sexual desire and psychophysiological changes that characterize the sexual response and cause interpersonal difficulty and marked distress.^3^ Several studies have demonstrated a high prevalence of sexual dysfunction in the general female population ranging from 30% to 49%.^4^,^5^ Sexual dysfunction significantly affects woman’s quality of life and self-esteem.^6^ Many risk factors associated with sexual dysfunction has been described in the literature, including postmenopausal status, long-term relationship with the partner, diabetes, pregnancy, alcohol consumption, nicotine use, pelvic organ prolapse (POP) and urinary incontinence (UI).^7^–^10^ Female sexual dysfunction (FSD) symptomsare ‘a departure from normal sensation and/or function experienced by a woman during sexual activity’ and has been categorized as sexual interest arousal disorder, orgasmic disorder and genito-pelvic pain/penetration disorder.^11^

It has been suggested that the pelvic floor muscle (PFM) tone, strength and ability to contract are significant factors for vaginal receptivity and responsiveness, for pleasure during intercourse for both partners, and for the orgasmic muscular response.^12^ Some studies have demonstrated that strong PFM may be related with better orgasmic and arousal potentials, desire, excitement and vaginal lubrication,^13^,^14^ in addition to enhanced vaginal sensation and tightness.^15^ Furthermore, voluntary PFM contractions improve pelvic blood circulation, triggering improvementsin sexual functioning.^16^,^17^ Moreover, it has been proven that the levatorani muscle contracts upon stimulation of the clitoris or cervix uteri, improving the sexual response.^17^ Thus, pelvic floor muscles both respond to sexual stimuli, and its voluntary contractions induce sexual reactions. To date, few studies exist regarding the relationship between the function of the pelvic floor muscles and female sexuality.^18^

Unlike the field of male sexual dysfunction, in which progress has been observed, the pathologic physiology, psychology and treatment of female sexual dysfunction are insufficiently elucidated as a result of the lack of a reliable diagnostic classification system and the limited number of studies performed. Several different factors determine this insufficiency of data on female sexuality. For this reason, in this study we aimed to determine PFM of women consulting at the gynecology outpatient clinics and its correlation with sexual dysfunction.

## METHODS

The study universe was represented by women who visited the gynecology outpatient clinics of the Inonu University Turgut Ozal Medical Center, the Malatya Public Hospital and the private hospitals in Malatya. The study was approved by the Regional Medical Ethics Committee and all subjects gave written informed consent before entering the study. Inclusion criteria was: (1) being over 18 years of age; (2) having good comprehension of verbal Turkish; (4) having a partner; and (5) being sexually active. Exclusion criteria wasgynecologic cancer or bladder cancer or kidney disease, diabetes mellitus, neurological disorders, alcohol consumption and nicotine use, prior treatment of urinary or bowel incontinence using biofeedback device or any other treatments, postmenopausal status, virginity and pregnancy.

The required sample size was calculated using the following formula, recommended for unknown universe size and prevalence: [n=(t 1-α)2 (p.q)2/S^2^]. The smallest sample size to remain in the 95% confidence interval found to be 384 subjects. The actual sample size was 430, using stratified groups. The data collection stage of the study was completed in three steps, namely preparation and implementation of the questionnaire, measurement of the pelvic floor muscle strength and data evaluation. The general information questionnaire, which was part of the data form, was designed to collect data on the participants, such as age, education status, occupation, monthly income, parity, delivery methods and body mass index (BMI). The questionnaires in this study included a general information form and an Index of Female Sexual Function (IFSF) worksheet. IFSF is an index developed by Kaplan *et al*.; it has been approved by the Turkish Society of Andrology and has been used in various investigations in Turkey and abroad.^19^

The IFSF was designed to evaluate different facets of female sexual function (lubrication, orgasm, desire, quality of sexual intercourse, clitoral sensation and overall satisfaction). Answers are evaluated on a scale from 1 (none or almost none) to 5 (always or almost). Subjects who have had no sexual intercourse in the last one month are attributed a zero score. The analysis of the individual questions is different from the scores for the different domains. Questions 1 and 2 inform about the quality of sexual intercourse, 4 and 5 about desire, 6 and 7 on quality of sexual intercourse, 8 on ability to achieve orgasm and 9 on clitoral sensation. The highest total score is 45, with a range of 5-45. A fall in the total score indicates a loss of sexual function. Even though the threshold value for sexual dysfunction in Turkey has not been determined, a score equal to or lower than 30 is interpreted as indicating the presence of sexual dysfunction.

The pelvic floor muscle strength of subjects was measured using a perineometer by one trained gynaecologists. The perineometer is a vaginal dynamometer that objectively assesses the strength of the pelvic floor musculature. A vaginal probe is advanced over about 3.5 cm and the patient is asked to contract her perineal muscles. The normal pressure range for a perineometer is 30-60 cm H_2_O. Values of 12-30 cm H_2_O are interpreted as low and those below 12cm H_2_O abnormally low.

The analysis of data was performed using the SPSS 16.0 software package. Arithmetic means were shown with 1 standard deviation. The chi-squared test, logistic regression and Pearson’s correlation analysis were used for the statistical evaluation.

## RESULTS

The mean age of the 430 participating women was 38.5±0.5, with a range of 20-50; 10.5% of them were illiterate. Housewifes represented 78.1% of the study sample. The monthly income was at or below the minimum wage level in 26% of participants. While 64.2% of participants lived in a group of five or more in the same lodgings, 73.0% were inhabited at a city (Table-I).

**Table-I:** Distribution of study subjects according to sociodemographic characteristics.

*Sociodemographic Characteristics*	*No.*	*%*
***Age Group (years)***		
20-29	116	27.0
30-39	130	30.2
40 or higher	184	42.8
***Education Status***		
Illiterate	45	10.5
Primary school	217	50.5
High School	120	27.8
Higher education	48	11.2
***Occupation***		
Housewife	336	78.1
Gainfully employed	74	17.2
Retired	20	4.7
***Income (Turkish Lira, YTL)***		
<700*	112	26.0
701-1401	164	38.1
1402-2102	100	23.3
2103>	54	12.6
***No. of Persons in Lodgings***		
4 or fewer	154	35.8
5 or more	276	64.2
***Settlement Type***		
Urban	314	73.0
Rural	116	27.0

Total	430	100.0

* Minimum wage for the year 2012.

The mean pelvic floor muscle strength of the participants were 31.4±9.6 cm H_2_O. The lowest value was 7 and the highest was 60 cm H_2_O. The pelvic floor muscular strength was measured lower than 12 cm H_2_O in 10% of the subjects; it was 12-30 cm H_2_O in 30.4% and 30-60 cm H_2_O in the remaining 59.6% (Table-II).

**Table-II:** Distribution of subjects by pelvic floor muscle strength.

*Pelvic Floor Muscle Strength Value*	*No.*	*%*
≤12 (very low)	43	10.0
>12 and ≤30 (low)	131	30.4
>30 and ≤60 (normal)	256	59.6

Total	430	100.0

Logistic regression analysis was performed to see the combined effect of independent variables found to be correlated with the pelvic floor muscle strength (Table-III).

**Table-III:** Factors affecting the muscular strength of the pelvic floor (logistic regression modeling).

*Independent variables*	*Regression coefficient B*	*Standard Error*	*p-value*	*Odds Ratio (OR)*	*%95 Confidence interval (CI)*
Parity	1.713	0.389	0.001	5.546	2.586-11.895
Age (>40)	1.248	0.288	0.0001	3.484	1.982-6.125

***Excluded from analysis:*** - Body mass index - Number of persons sharing same lodgings - Occupational status - Educational status - Income status - Settlement type.

Parity (odds ratio OR=5.546, confidence interval CI=2.586-11.895) and age 40 or higher (OR=3.484, CI=1.982-6.125) were primary factors correlated with pelvic floor muscle weakness (p<0.05).

The mean IFSF score in this study was 26.5±6.9. The lowest IFSF score was 5 and the highest was 45. The IFSF score was lower than 30 in 69.1% of all subjects and 30 or higher in 30.9% (Table-IV).

**Table-IV:** Distribution of subjects according to IFSF scores.

*IFSF Score*	*No.*	*%*
<30	297	69.1
≥30	133	30.9

Total	430	100.0

Logistic regression analysis was performed to see the combined effect of independent variables found to be correlated with the IFSF score; the results are shown in Table-V. The factors directly correlated with sexual dysfunction were being overweight (OR=2.105, CI=1.303-3.399) and age 40 or higher (OR=2.451, CI=1.578-3.809) (p<0.05).

**Table-V:** Factors affecting IFSF score (logistic regression modeling).

*Independent variables*	*Regression coefficient B*	*Standard Error*	*p-value*	*Odds Ratio (OR)*	*%95 Confidence interval (CI)*
Body mass index (overweight)	0.744	0.245	0.002	2.105	1.303-3.399
Age (over 40)	0.897	0.225	0.001	2.451	1.578-3.809

***Excluded from the analysis:*** - Educational status - Parity number - Delivery method.

IFSF scores were under 30 in 64.1% of patients with pelvic floor muscle strength in normal limits,78.9% of patients with pelvic floor muscle strength in low limits (>12 and ≤30) and 72.1% of subjects with very low muscular strength (≤12). The distribution of the IFSF scores were found statistically significantly different according the pelvic floor muscle strength values (p<0.05). Low pelvic floor muscle strength was found responsible for this difference(Table-VI).

**Table-VI:** Distribution of IFSF scores according to the pelvic floor muscle strength.

*Pelvic Floor Muscle Strength*	*IFSF Score*				*Total*	

	*<30*		*≥30*			

	*Number*	*%*	*Number*	*%*	*Number*	%*
>30 and ≤60	164	64.1	92	35.9	256	59.6
>12 and ≤30	102	78.9	29	21.1	131	30.4
≤12	31	72.1	12	27.9	43	10.0

Total	297	69.1	133	30.9	430	100.0

* ***Column percentage in this column***

p< 0.05 χ^2^ = 7.929 SD=2 p< 0.05 χ^2^ = 7.425 SD=1(Second row excluded).

Pearson’s correlation analysis showed a statistically significant, linear correlation between the muscular strength of the pelvic floor and sexual function, with an r coefficient of 0.196. Albeit weak, this linear correlation was significant (p=0.001).

## DISCUSSION

There are a few studies about the use of perineometer for assessing pelvic floor muscle strength in the literature. In a study that measured this strength using a vaginal manometer in both incontinent and continent women, pressure values under 12 cm H_2_O were deemed abnormal.^20^ In this study while the pelvic floor muscular strength was measured lower than 12 cm H_2_O in 10% of the patients; it was measured 12-30 cm H_2_O in 30.4%. The available literature indicates that the pelvic floor muscles become weaker Following each delivery Vaginal delivery has been considered the most significant factor for pelvic floor muscular weakness. A prolonged second phase of delivery has been hypothesized for pelvic floor muscle injury. Authors called attention to the fact that the mode of delivery has not been shown to correlate with the muscular strength of the pelvic floor over the long term.^21^,^22^ The present study demonstrated that parity and being age 40 or over were correlated with the pelvic floor muscle strength consistent with the literature.

Female sexual function is an important element of quality of life. Sexual dysfunction is a widespread, age-related, progressive problem afflicting 30-50% of women.^23^ In this study the sexual function of 430 women aged 20-50 were evaluated by IFSF questionnaire and IFSF scores were recorded. Even though the threshold value for sexual dysfunction in Turkey has not been determined, a score equal to or lower than 30 is interpreted as indicating the presence of sexual dysfunction.^19^ We observed that overall mean IFSF score was 26.9±7.8 and 69.1% of the subjects had sexual dysfunction in our study. While data on the incidence and prevalence of female sexual dysfunction are rather scarce in published literature, some authors reported substantially high figures reaching 76%.^24^ The study with the largest series was published in the US by Laumann *et al*. The sexual dysfunction rate among 1749 women aged 18-59 was reported 43%. Besides, the sexual dysfunction rateshave been reported 33% and 22% respectively.^25^ The variation in prevalence rates across the publications may be due to differences in the definition of sexual dysfunction and the research methods, and also to the sociocultural structure of the different societies in which the studies were conducted.

Age has been designated as the most important factor affecting sexual function. Numerous physiological, psychological and social factors, such as the age-related deterioration of the functional capacity of tissues and organs, increasing genital organ and pelvic floor muscle dysfunction, structural and hormonal change due to pregnancy and delivery, increased frequency of chronic disease and the weight of sociocultural value judgments are believed to negatively affect female sexual function.^26^ In this study, being age 40 or over was found primary factor with pelvic floor muscle weakness (p<0.05). Published reports showed that the prevalence of female sexual dysfunction increases along with age. The increased sexual dysfunction with advancing age found in this study was consistent with the available literature.

In this study, IFSF scores were under 30 in 64.1% of subjects whose pelvic floor muscle strength was within normal limits, 78.9% of those with low pelvic floor muscle strength and 72.1% of subjects with very low muscular strength. Pearson’s correlation analysis showed a statistically significant, direct linear correlation between the muscular strength of the pelvic floor and good sexual function. A survey of the literature shows only few studies demonstrating the relationship between PFM strength and sexual dysfunction were performed. Similarly Zaharaiou et al demonstrated thatwomen with weak muscles who receive pelvic floor muscle training strengthen the muscles in this region noticed a positive effect on their sexual life. They suggested thatpelvic floor muscle contraction plays an important role in the female orgasmic response. Furthermore, the strength of pelvic floor muscles probably affects the anatomical position of clitoral erectile tissue with consequences to sexual stimulation.^27^ Although most studies indicated a significant improvement in sexual dysfunction after pelvic floor muscle training between control and intervention groups, Ferreira et al suggested that the results need to be interpreted with caution and high-quality RCTs specifically designed to investigate the impact of pelvic floor muscle training on women’s sexual function are required.^28^

## CONCLUSION

We have demonstrated that the mean IFSF score was higher in the patients with normal pelvic floor muscle strength. We observed that higher age and higher body mass index was associated with higher sexual dysfunction. By being aware of these predictors, clinicians were able to emphasize additional benefits for sexual dysfunctionwith interventionsthat improving pelvic floor muscle strength like doing pelvic floor muscle exercise. Furthermore, we believe that asking women about sexual problems in routine gynecology practice would be useful to decrease the threshold for women experiencing these problems by giving chance them consider about their problems and treatment options with their physicians.

### Authors Contribution

**FCO:** Project development, Data Collection, Manuscript writing.

**EP:** Data collection.

**RM:** Manuscript writing.
